# Fatal Fast-Evolution of Nasopharyngeal Squamous Cell Carcinoma in an HIV Patient with EBV and HPV (-16 AND -33) in Blood Serum

**DOI:** 10.2174/1874613600802010001

**Published:** 2008-02-06

**Authors:** Guillem Sirera, Sebastià Videla, Joan Romeu, MariPaz Cañadas, Maria-Teresa Fernández, Susana Balo, Beatriz Cirauqui, Laila Darwich, Celestino Rey-Joly, Bonaventura Clotet

**Affiliations:** a HIV Clinical Unit, Department of Medicine; b Lluita Contra La SIDA Foundation; d Department of Pathology; e Department of Oncology; University Hospital Germans Trias i Pujol, Badalona (Barcelona); Universitat Autònoma de Barcelona (U.A.B.); c GeneralLab, Barcelona; f Retrovirology Laboratory IrsiCaixa, Spain

## Abstract

Our case illustrates the first report of an HIV-infected patient with a nasopharyngeal squamous cell carcinoma with viremia by one Epstein-Barr virus (EBV) and seropositivity by two high risk oncogenic human papilloma viruses (HPV)-types (HPV-16 and HPV-33), previous to his death. This patient presented a fatal fast-evolution.

## CASE REPORT

**Fig. (1):**
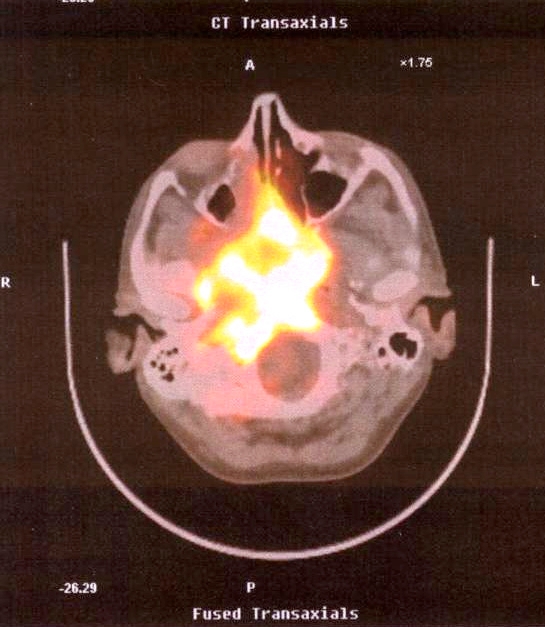
P.E.T. showed a hypermetabolic mass from the skull base to the right jawbone.

A 38 year-old man diagnosed with HIV in 1987 (disseminated tuberculosis) and HCV, who was an ex-IDU, smoker (30 cigarettes per day), and alcohol consumer (30 g/day), with poor HAART adherence, and a CD4 nadir of 6 cells/mm^3^ in 2005, was seen on April 20^th^ 2006 in a routine HIV control. He reported having an oppressive headache in the last week. The exploration only showed hypoesthesia in the right side of the face. A small ulcer in the cavum and an exophytic polypoid lesion in the back and upper area of the right nostril were observed by nasopharyngoscopy. Computed tomography visualized a mass in the cavum. A biopsy diagnosed a moderately differentiated squamous cell cancer. P.E.T. showed a hypermetabolic mass from the skull base to the right jawbone (Fig. **1**). In addition, P.E.T. also showed infiltration in the latero-cervical, supra clavicular and para-oesophagus nodes, in C3 and C6 (cervical vertebras), and in the liver (not shown). The patient restarted HAART on May 9^th^ and was discharged. The patient was readmitted to the hospital on June 6^th^ and he died one week later because of liver failure. The patient signed the informed consent to study HPV (June 6^th^) and his family authorized to publish this event.

Epstein-Barr Virus (EBV) and Human Papilloma Virus (HPV) were studied in biopsy and in blood serum sample (June 6^th^) by PCR real time and multiplex PCR kit, respectively. Furthermore, HPV was analyzed in samples from the oral cavity, anus, and penis. EBV was positive in the biopsy and in serum (63000 copies/mL) and HPV was positive in serum for two high risk oncogenic types: HPV-16 and HPV-33. HPV was also detected in the anus (HPV-16 and HPV-51) and in the penis (HPV-51).

We believe that our case illustrates the first report of an HIV-infected patient with a nasopharyngeal squamous cell carcinoma with a viremia by one EBV and two high risk oncogenic HPV-types, previous to his death. Although EBV appears to be the most important etiological factor for the

development of nasopharyngeal cancer and its presence in serum suggests metastasis [1,2], the HPV has been also implicated as an etiological factor in a subset of head and neck squamous cell carcinomas (HNSCC), and its presence in the sera has been related with an advanced HNSCC stage, or with a high risk for metastasis and with a poor prognosis [3-5]. Although we did not find HPV in the biopsy, we cannot dismiss its presence [3]. Moreover, he was HPV seropositive which could mean that the tumor was the source for it. The serum sample studied could only represent a single time point during the patient’s care, however HPV DNA in serum may represent occult haematogenous spread of cancer cells. So, it may be acceptable that the origin of the serum HPV could be the tumor.

Patients with HPV-positive tumors have a better overall survival than patients with HPV-negative tumors [4]. It has been observed that a normal host immunity may block metastatic disease [3]. So, the rapid evolution observed could be mainly related to the severe immunodeficiency HIV related. Indeed, the widespread metastatic lesions suggest the expression of chemokine receptor CXCR4 which has been associated with a high rate of metastatic potential of human nasopharyngeal carcinoma [6]. Likewise, other risk factors such as being smoker and an alcohol consumer could contribute to the aggressive clinical outcome as well.

In summary, this case suggests that in very advanced HIV patients with severe immunodeficiency, tumors may have a rapid evolution and different viruses etiologically related (HPV and EBV) can be detected in blood which could be associated with a worse and rapid outcome.

## CONFLICT OF INTEREST

S.V. has received honoraria for collaborating with Laboratorios Dr Esteve. B. Clotet has received honoraria for speaking and participating in advisory boards from Abbott, Bristol-Myers Squibb, Boehringer-Ingelheim, Gilead Sciences, GlaxoSmithKline, Pfizer.
